# Major Role of Multiscale Entropy Evolution in Complex Systems and Data Science

**DOI:** 10.3390/e26040330

**Published:** 2024-04-12

**Authors:** Shahid Nawaz, Muhammad Saleem, Fedor V. Kusmartsev, Dalaver H. Anjum

**Affiliations:** 1 Department of Physics, Loughborough University, Loughborough LE11 3TU, UK; 2 Department of Physics, Bellarmine University, 2001 Newburg Road, Louisville, KY 40205, USA; 3 Department of Physics, Khalifa University, Abu Dhabi P.O. Box 127788, United Arab Emirates

**Keywords:** complex system, multiscale entropy dynamics, generalized nonlinear Schrödinger equation, data science, quasiparticle

## Abstract

Complex systems are prevalent in various disciplines encompassing the natural and social sciences, such as physics, biology, economics, and sociology. Leveraging data science techniques, particularly those rooted in artificial intelligence and machine learning, offers a promising avenue for comprehending the intricacies of complex systems without necessitating detailed knowledge of underlying dynamics. In this paper, we demonstrate that multiscale entropy (MSE) is pivotal in describing the steady state of complex systems. Introducing the multiscale entropy dynamics (MED) methodology, we provide a framework for dissecting system dynamics and uncovering the driving forces behind their evolution. Our investigation reveals that the MED methodology facilitates the expression of complex system dynamics through a Generalized Nonlinear Schrödinger Equation (GNSE) that thus demonstrates its potential applicability across diverse complex systems. By elucidating the entropic underpinnings of complexity, our study paves the way for a deeper understanding of dynamic phenomena. It offers insights into the behavior of complex systems across various domains.

## 1. Introduction

Probability theory is a common thread that binds the fields of statistical physics, information theory, and data science in sharing standard mathematical and conceptual foundations. These are interconnected in several ways [1,2] and deal with the analysis of complex systems and use similar mathematical and statistical tools to model and analyze these systems. One of the most critical connections between these fields is the concept of entropy [3]. In statistical physics, entropy measures disorder or randomness in a physical system and can be used as a main driving force for the second law of thermodynamics [4]. Entropy measures a dataset’s uncertainty or information content in information theory. Quantum mechanics is conventionally formulated using two conjugate variables that obey Heisenberg’s uncertainty principle in the Hilbert space. For instance, the momentum *p* and the position coordinate *q* in the form of ΔqΔp≥ℏ/2. This formulation of Heisenberg’s principle describes the statistical nature of these self-adjoint operators $\hat{q}$ and $\hat{p}$ in the Hilbert space. The ED formulation of quantum mechanics was introduced in 2009 and has been applied to fields such as quantum measurement problems [5,6], uncertainty relations [7], curved space–time [8], scalar fields [9,10,11], and finance [12]. The ED approach is an alternative formulation of QM, in which the dynamics of a probability distribution are derived from the entropy [13,14]. This approach describes the discord between the quantities’ dynamics and probabilistic nature more conveniently than other approaches. In the entropic dynamics (ED) formulation, the quantum nature of these operators is given a secondary role. In fact, in this approach, the uncertainty in these variables stems from the diffusion process of the Brownian motion of the particles [7,15]. It is important to note that momentum is not real in ED formulation but is an epistemic property of wave functions and not a property of the particles. In classical mechanics, we assume that the particles’ positions and their momenta are real. In the ED approach, only the positions are real because ED does not describe particle dynamics but only their probabilities. Another important fact of the ED formulation is that no conventional momentum is canonically conjugated to the generalized coordinates. As we mentioned above, the generalized coordinates represent the probabilities, not the actual positions. Thus, there is no momentum for the particles in the ED formulation. Of course, we can always consider translations, and following convention, we can call the generator of translations “linear momentum”. But this is just a name for operators that are not properties of the particles.

Understanding complex systems is a multidisciplinary field that extracts valuable insights and knowledge from structured and unstructured data. The nature of unstructured data must be understood before applying data science techniques to gain physical insights from the data. Using diverse techniques, methods, and tools from statistics, mathematics, computer science, and domain-specific knowledge, data science’s roots trace back to data collection, which has become a ubiquitous aspect of nearly every human activity over time. As computer facilities have advanced, the ability to gather relevant data from various sources has created extensive databases. Consequently, ensuring the quality and quantity of collected data has become paramount, prompting a detailed analysis to investigate their impact directly. Exploratory Data Analysis (EDA) plays a crucial role in this process, involving thorough examination, analysis, and data visualization. Its objective is to understand the data’s patterns, trends, and relationships, facilitating the construction of predictive models through algorithms that learn from the data at hand.

One crucial aspect of data science is feature data engineering, which involves predicting the evolution of data flow. This process incorporates big data technologies capable of handling and processing large volumes of data. The field of data science is rapidly evolving, which requires practitioners to stay up to date on new techniques and technologies. In this dynamic and interdisciplinary field, finding and implementing models that can perform fast and efficient analyses and provide realistic predictions over time is essential. Considerations such as scalability, real-time processing, and model drift are integral to the practice of data science. Practitioners often specialize in specific areas based on their interests and expertise. The application of data science is extensive, spanning various industries, including healthcare, finance, marketing, and technology. In simple terms, the application of data science to a database of a complex system demands the organization of data into numerous sets, with each mathematical set comprising data identified by their physics-dictated similarities. It is to be noted that the concept of similarity in datasets of complex systems is broad, encompassing scenarios such as sets containing snapshots of cars crossing red lights at road intersections or exceeding speed limits in specific locations of special importance. Consequently, we obtain multiple sets, allowing for the identification of the probability of traffic rule violations, denoted $p_{i}$, and the estimation of the information entropy describing the information content. The first probability ($p_{i}$) represents the ratio of elements in a particular set to the total number of elements in all sets.

Using probability functions, the next step involves estimating the Shannon information entropy, denoted $S_{p}$. This marks the initial phase. Subsequently, in the second step, we need to consider the value of police fines established for each of these traffic rule violations. The fines for the *i*th type of violation occur with the probability $p_{i}$ and have the value $E_{i}$. Therefore, these fines can be incorporated into the entropy expression using Lagrange multipliers, e.g., a constant β. By determining the maximum of the total entropy (or information content) function equal to $S_{total}=S_{p}\left(p_{1},\ldots ,p_{N}\right)+\beta \sum_{i}p_{i}E_{i}$, we find the initial entropy or content information in the existing dataset. Here, the function $S_{p}\left(p_{1},\ldots ,p_{N}\right)$ is expressed as Shannon entropy and measures the uncertainty or information content in a dataset. It is calculated using the formula:

(1) $$S_{p}=-\sum_{i=1}^{N}p_{i}\log_{2}\left(p_{i}\right)$$

where $p_{i}$ represents the probability of the occurrence of the *i*th event described the a certain set of data. In the context of the provided text, this entropy measure is applied to assess the information content associated with traffic rule violations.

Here, we may reveal the dependence of each $p_{i}$ on the value of the fine $E_{i}$ in a manner very similar to what is used in statistical mechanics at the derivation of the Boltzmann distribution. With this step, the analysis of the existing datasets is concluded, and the probability distribution of police fines is derived. In the subsequent second stage, our objective is to find or predict the evolution of this complex system. Here, we used an analogy between the cost of the fine and the energy in statistical analysis; see Ref. [16].

It is well established that in any complex system in physics, e.g., classical and quantum gases, the entropy of any initial equilibrium state increases with time during interactions between particles. This fundamental observation constitutes the cornerstone of the second law of thermodynamics, with many confirmations. Naturally, this prompts the assumption that the evolution of entropy, particularly its growth, extends far beyond the conventional subject of physics. Thus, in the second stage of our approach, our primary objective is to derive equations for the time evolution of the entropy in any complex system. The evolution of entropy begins with the initial entropy calculated from the datasets in the first stage of our approach. In numerous cases this paper considers, the equations governing entropy evolution are reduced to various nonlinear Schrödinger equations. This is noteworthy, especially considering that traditional data science methods have been predominantly used, such as the multidimensional version of gradient descent. While such methods prove useful in many cases, they face challenges in realistic systems due to their multidimensional nature. For instance, introducing the system’s internal energy concept, represented as an integral function of entropy, reveals a complex energy landscape with numerous minima and maxima. The multidimensional gradient descent method may lead the system to a false minimum or maximum. An alternative and more advanced approach involves machine learning. It improves the situation by training the evolution of entropy on the initial time steps and then extrapolating it for all subsequent times. However, the approach proposed in this paper offers a comprehensive formulation that can be applied to any database.

We propose using specific equations to describe the dynamics of entropy, which is found to depend on the characteristics of the system under consideration. In the context of the information provided by data science, the entropy evolution may be described by nonlinear Schrödinger equations (NLSEs). It is a partial differential equation that commonly appears in various areas of physics, including optics, plasma physics, and condensed matter physics. Its general form is:

(2) $$i\hbar \frac{\partial \psi }{\partial t}=-\frac{\hbar^{2}}{2m}\frac{\partial^{2}\psi }{\partial x^{2}}+V\left(x\right)\psi +g_{n}{\left|\psi \right|}^{2}\psi$$

where ψ represents the wave function, *t* is time, and *x* is the coordinate of the generalized spatial position, as in classical mechanics. *ℏ* is the reduced Planck constant, *m* is the particle mass, V(x) is the potential energy, and the constant $g_{n}$ characterizes the strength of the nonlinearity. This equation describes the evolution of the wave function ψ(t) over time, and its nonlinearity term $g_{n}{\left|\psi \right|}^{2}\psi$ is a key feature that distinguishes it from the linear Schrödinger equation. In the context of the provided information, the use of nonlinear Schrödinger equations suggests that the evolution of entropy in certain complex systems follows a mathematical framework akin to that of quantum mechanics. The specifics of how these equations are adapted or derived for entropy evolution in data science applications require a more detailed understanding of the particular system and the associated mathematical modeling choices.

The presented methodology of multiscale entropy evolution (MEE) can be applied to any complex system and is useful in data science. The details of the methodology, such as the concepts and main framework, are given in the next section. There, the goal of our approach is to find entropic dynamics, which represent the evolution function of information contained in data. The paper is organized as follows: In Section 2, the main concepts and general framework of the multiscale entropy (MSE) and its evolution, i.e., multiscale entropy evolution or dynamics (MEE or MED), are presented. Section 3 provides mathematical concepts of the MED formulation and framework. It also delineates the MED formulation of QM, which is briefly presented. The approach to complex systems using MED is discussed in Section 4. We demonstrate how the MED framework can be used to derive the Generalized Nonlinear Schrödinger Equation (GNSE). In Section 5, the derived GNSE is applied to quasi-particle dynamics (e.g., plasmons) or solitons and other nonlinear phenomena. Furthermore, a form for interacting electromagnetic fields with solitons is also presented. The effect of temperature and pressure on the evolution of the complex system of quantum particles is presented in Section 6. The ramifications of the presented results are discussed at length in Section 7. In this section, we present reasons that the relation of MED-determined quantities can be applied to data science. The overall conclusions drawn from this study are presented in the last conclusions Section 8.

## 2. Main Concepts and General Framework

Here, below, we outline the main concepts of multiscale entropy (MSE) and its evolution, MEE, and the general framework for their broad applications.

### 2.1. The Concept of Multiscale Entropy (MSE)

We start by defining entropy as a measure of disorder or uncertainty in a system, highlighting its importance in various fields such as physics, information theory, and economics. In information theory, it is a measure of the amount of information. This is usually described by the Shannon entropy equation, a generalization of the Boltzmann Entropy associated with the number of microstate configurations. Multiscale entropy is a method to analyze complexity and irregularity at different scales or levels of a system [17,18]. Entropy is a fundamental concept in thermodynamics and information theory that quantifies the degree of disorder or randomness of a system. In thermodynamics, entropy is related to the number of microstates associated with a macroscopic state of a system, reflecting the system’s tendency to evolve towards equilibrium. In information theory, entropy measures the uncertainty or content of a random variable. The entropy of a system is typically indicated by *S* and is defined by the Boltzmann formula:

(3) S=klnW

where:

- *k* is the Boltzmann constant ($1.38\times 10^{-23}$ J/K in SI units);
- *W* is the number of microstates corresponding to the macroscopic state of the system;
- *ln* represents the natural logarithm.

Entropy increases in a closed system over time, reflecting the trend toward increasing disorder or randomness. Multiscale entropy (MSE) is an extension of entropy analysis that considers entropy on different time scales or resolutions [19]. It is particularly useful for studying complex systems with varying dynamics on multiple temporal scales. The concept of MSE involves calculating entropy measures at different scales and examining how entropy changes with scale. The general concept of multiscale entropy (MSE) can be represented mathematically as follows [17,20]:

1. Calculate the sample entropy $S^{m}$ for a time series x(t) at a specific scale *m*.
  (4) $$S^{m}=ln\;C^{m}\left(r\right)$$
  where $C^{m}\left(r\right)$ is the conditional probability that two sequences of length m+1 with distance less than *r* are similar.
2. Calculate the average entropy on all scales from m=1 to *M*, where *M* is the maximum scale.
  (5) $$MSE=\frac{1}{M}\sum_{m=1}^{M}S^{m}$$

The MSE is a mathematical concept that characterizes the diversity or variability observed in different scales within a system and offers insight into how emergent properties influence all subsystems of the system. Emergent properties refer to novel behaviors or patterns arising from individual components’ interactions within a complex system. In the context described, strong emergent properties manifest themselves as oscillations of a multiscale variety, sometimes exhibiting negative values, a distinctive feature of such phenomena [17]. These emergent properties often have significant implications for the behavior of the system and have numerous applications [20]. An example of the relevance of multiscale entropy in social systems lies in understanding various allocation, optimization, and functional requirements that govern system behavior. These could include resource allocation strategies, process optimization, or functional requirements that shape individuals or groups within the system [19]. Multiscale complex system analysis, particularly multiscale entropy evolution, may improve the effectiveness of healthcare and public health [21]. Investigation of memory consolidation across different age groups found that multiscale entropy (MSE) is a sensitive measure to improve memory mechanisms [22]. The MSE measures can distinguish sleep stages in preterm infants [23,24]. Jelinek et al. applied multiscale Renyi entropy on HRV, highlighting its efficacy in detecting differences between disease classes [25]. El-Yaagoubi et al. have studied MSE evolution (MEE) over long periods, revealing significant differences between heart conditions [26]. The MSE concept helped to develop an entropy-based structural health monitoring system, demonstrating the utility of MSE in assessing structural damage [27]. Furthermore, strong emergent properties suggest a causal relationship that operates from the global to the local level. This concept challenges traditional scientific notions that adhere to a strict local-to-global causality framework. Although this conceptual change may initially be unsettling, it offers valuable information on the dynamics of biological and social systems. Understanding and incorporating these global-to-local causal relationships is crucial to developing more comprehensive models of complex systems, contributing to a deeper understanding of their behavior and dynamics and making a realistic diagnosis. For example, using the MSE concept, Ge et al. proposed a bearing failure diagnosis technique that employs robust local principal component analysis and multiscale permutation [28]. At the same time, Perpetuini et al. utilized fNIRS MSE to diagnose early Alzheimer’s disease, showing promising diagnostic capabilities [29].

The MSE algorithm involves several steps, such as the following:

1. Divide the time series x(t) into non-overlapping segments of length *m*.
2. Calculate the sample entropy $S^{m}$ for each segment at scale *m*.
3. Average the sample entropy values over all scales to obtain the multiscale entropy.

This approach allows for the analysis of the entropy of complex systems in stationary states with no temporal resolution; therefore, it cannot reveal patterns and complexities that may be apparent when considering entropy on a single scale. Therefore, the MSE can be defined as a composite measure that reflects the overall complexity of a time series signal across multiple scales. It is obtained by averaging the sample entropy values on all scales from m=1 to *M*.

### 2.2. The Concept of Multiscale Entropy Evolution (MEE and MED)

On the other hand, the multiscale entropy evolution (MEE) or MED approach involves computing sample entropy at various scales and time domains, averaging these entropies to obtain MSE [17,18,19]. The importance of MSE and MEE has already been shown in numerous examples. Thus, Keshmiri et al. examined human activity using MSE, indicating increased complexity with physical embodiment, reflecting perceived fatigue [30]. Xu et al. analyzed the complexities of Wikipedia page views with short-time series MSE (sMSE), providing insights into human website search behaviors [31]. Shang et al. introduced a novel feature extraction method for partial discharge fault analysis utilizing variational mode decomposition and multiscale dispersion entropy, showcasing the versatility of MSE in fault analysis applications [32]. The framework presented here allows researchers to study how complexity and irregularity change over different time scales, providing valuable insights into the behavior of complex systems. We show that such evolving system dynamics can be analyzed using GNSE or similar linear or nonlinear equations in the general case, which can be written as:

(6) $$\hbar \frac{\partial }{\partial t}\psi =\hat{H}\psi$$

where:

- ψ represents the system’s wave function.
- $\hat{H}$ is the Hamiltonian operator representing the system’s energy operator, which, in general, may depend on ψ.
- *ℏ* is the reduced Planck constant.
- *t* is time.

### 2.3. General Concept of Complex Systems

A complex system can be defined as a system characterized by the presence of multiple interacting components or agents, often exhibiting emergent properties and nonlinear behavior [33]. Examples of complex systems in various domains include the following. **1. Biological networks:** Biological systems such as gene regulatory networks, metabolic pathways, and neural networks are highly complex. The interactions between genes, proteins, and other biomolecules give rise to emerging properties, such as robustness, adaptability, and the ability to evolve. **2. Ecological Systems:** Ecosystems are complex networks of interactions between living organisms and their environment. They involve intricate food webs, nutrient cycles, predator–prey relationships, and feedback mechanisms. Changes in one part of an ecosystem can have cascading effects throughout the system. **3. Social Networks:** Social systems, including social media networks, organizational structures, and human societies, are complex due to the diverse and dynamic interactions between individuals, groups, and institutions [34]. These interactions lead to information diffusion, opinion formation, collective behavior, and social dynamics. **4. Financial Markets:** Financial systems are complex networks of interactions between investors, financial institutions, assets, and economic factors. The behaviors of market participants, coupled with factors like risk perception, market sentiment, regulatory changes, and global events, contribute to the complexity of financial markets. This complexity can lead to market bubbles, crashes, and systemic risks. Understanding and analyzing complex systems requires approaches that consider the system as a whole rather than focus solely on its components. Techniques such as network theory, associated agent-based modeling, and network system dynamics play crucial roles in studying and modeling complex systems in diverse disciplines [33].

### 2.4. The Main Framework of Application of MSE, MEE, and MED

Just like the case of MSE, the application of MEE and MED methodology to analyze and model complex systems also involves several steps and strategies. Here is a general description of how MED can be applied. **1. Data Collection and Preprocessing:** The first step is to gather data related to the complex system under study. This could be time series data, network data, or any other relevant data format. To prepare for analysis, data may need pre-processing, such as normalization, filtering, or feature extraction. **2. Scale selection:** Determine the appropriate scales or levels of observation for the analysis. This involves deciding the range of scales (from fine to coarse) to compute entropy measures. **3. Entropy Calculation:** Calculate entropy measures at each selected scale. This typically involves computing Sample Entropy (S) or other entropy measures for different window sizes or resolutions across the data. **4. Multiscale Entropy (MSE):** Compute multiscale entropy (MSE) by averaging the entropy values across all selected scales. MSE provides a comprehensive measure of complexity and irregularity across multiple temporal scales. **5. Pattern Analysis:** Analyze patterns and trends in the MSE values. Look for changes in complexity over time or across different scales, which can reveal important dynamics within the complex system. **6. Modeling and Simulation:** Use the insights from MED to develop models or simulations of the complex system. This may involve creating mathematical models based on the observed entropy dynamics or using computational simulations to simulate system behavior. **7. Validation and Interpretation:** Validate the models or simulations against real-world data to ensure their accuracy and reliability. Interpret the results in the context of the complex system’s dynamics, patterns, and interactions. **8. Application to Real-World Problems:** Apply the MED approach to real-world problems or scenarios related to the complex system. This could include predicting future trends, identifying anomalies or critical events, or optimizing system performance based on entropy dynamics. The MED method proposed in the paper provides a systematic framework for analyzing the dynamics, patterns, and interactions within complex systems by leveraging entropy measures across multiple scales. Combining mathematical analysis, statistical techniques, and computational modeling allows us to understand the underlying mechanisms that drive complex system behavior.

### 2.5. Advantages of MEE Approach

There are several advantages to using MEE and MED to study complex systems and their importance in data science and interdisciplinary research. For instance, below, we will show that the MED approach can capture the multiscale nature of complexity, identify patterns, detect anomalies, and provide insights into system behavior. In this way, the MED approach offers several advantages when applied to studying complex systems, making it a valuable tool in data science and interdisciplinary research in the following areas. **1. Multiscale Nature of Complexity:** One of the key advantages of MED is its ability to capture the multiscale nature of complexity within a system. By analyzing entropy measures across different temporal scales or levels of observation, MED can reveal how complexity manifests and evolves at various resolutions. This is crucial for understanding complex systems where phenomena occur at multiple scales simultaneously. **2. Pattern Identification:** MED enables the identification of patterns and structures within complex systems. Analyzing entropy dynamics across scales makes it possible to detect recurring patterns, trends, and regularities that may not be apparent on a single scale. This helps researchers uncover the underlying dynamics and relationships within the system. **3. Anomaly Detection:** MED’s advantage is its ability to detect anomalies or deviations from expected behavior within complex systems. Sudden changes in entropy measures on all scales can indicate the presence of anomalies, critical events, or unexpected changes in system dynamics. This ability is valuable for early warning systems and anomaly detection algorithms. **4. Insights into System Behavior:** By studying entropy dynamics using MED, researchers gain valuable insights into the behavior of complex systems. Changes in entropy over time and across scales provide clues about the stability, resilience, phase transitions, and emergent properties of the system. This deeper understanding helps to make informed decisions and design control and system management interventions. **5. Interdisciplinary Research:** MED bridges the gap between disciplines by providing a common framework for analyzing complex systems. It allows researchers from diverse fields, such as physics, biology, economics, and social sciences, to collaborate and gain insight into complex phenomena using a unified approach. This interdisciplinary nature of MED fosters the cross-pollination of ideas and accelerates innovation. **6. Data Science Applications:** In data science, MED plays a significant role in the analysis of large-scale datasets and the extraction of meaningful information. Its ability to capture multiscale complexity makes it suitable for processing complex and high-dimensional data, such as time series, network, and spatial data. MEE- or MED-based analyses contribute to the advancement of data-driven decision-making and predictive modeling. MED offers a powerful framework for studying complex systems, providing a holistic view of system dynamics, uncovering hidden patterns, detecting anomalies, and facilitating interdisciplinary research and data science collaboration. Its importance lies in its ability to handle the inherent complexity of real-world systems and extract actionable insights for various applications.

### 2.6. The Major Benefits of the Method

Some selected examples that follow show how MED can be applied to different complex systems, namely biological networks, climate models, financial time series, and social dynamics. **1. Biological networks** are the first example of the analysis of protein–protein interaction networks. MED can potentially reveal the hierarchical organization and modularity of these networks, identifying key protein clusters and their roles in cellular functions. This helps to understand the propagation of signals, resilience to perturbations, and disease mechanisms. In this way, the challenge of capturing dynamic changes in network topology, quantifying information flow across scales, and integrating omics data for comprehensive analysis can be addressed using MED in identifying biomarkers, drug targets, and regulatory pathways, advancing personalized medicine and system biology. **2. Climate models** study climate variability and extreme weather events. The MED approach can uncover multiscale temperature, precipitation, and atmospheric dynamics patterns, elucidating the drivers of climate change, El Niño/La Niña phenomena, and regional climate impacts. MED helps integrate observational data with climate models, quantify uncertainty, and predict long-term trends and abrupt shifts. Therefore, it improves climate projections, informs adaptation strategies, and improves risk assessment for climate-related hazards. **3. Financial time series** are used to analyze stock market fluctuations and risk management. Again, the MED approach can detect temporal correlations, volatility clusters, and regime changes in financial data, highlighting market trends, investor behavior, and systemic risk factors. In this application, the MED approach can model nonlinear dependencies, handle high-frequency data, and mitigate market anomalies and bubbles. Therefore, MED-driven analyses inform algorithmic trading strategies, portfolio optimization, and early warning systems for financial crises. **4. Social networks** control social dynamics and information diffusion, which can be handled using the MED approach. MED can reveal community structures, influence propagation patterns and sentiment dynamics on online platforms, and facilitate understanding of social trends, polarization phenomena, and spreading misinformation. In this way, dynamic interactions are modeled, user engagement patterns are captured, and privacy concerns and ethical considerations are addressed. MED-based studies can inform digital marketing strategies, policy interventions for online platforms, and crisis communication strategies. Through these examples, MED demonstrates its versatility in the analysis of diverse complex systems, providing valuable information, addressing specific challenges, and contributing to a deeper understanding of complex phenomena in various domains. By adopting this structured approach, the paper can provide a clearer and more comprehensive understanding of how multiscale entropy dynamics is applied to complex systems, avoiding the fragmentation of examples and ensuring a unified conceptual framework for readers to grasp the methodology and its implications.

### 2.7. Illustrative Applications of the Method Framework

Finally, we would like to describe how to utilize the multiscale entropy (MSE) evolution method for data science in the context of the Chinese carbon market, which is vital for the climate change of the Earth [35]. Here, we can follow these steps: First, we must perform data collection by gathering relevant data on carbon emissions, trading volumes, prices, and market behavior from the pilot markets in China. Then, we must perform data preprocessing by cleaning and preprocessing the data to ensure accuracy and reliability. This may involve handling missing values, removing outliers, and normalizing data if necessary. In the next step, we must apply MSE to the preprocessed data to assess the complexity of the Chinese carbon market across different time scales. This analysis will provide insights into the patterns and dynamics of market behavior. Next, we utilize the moving average method for scale extraction [35], considering the limited dataset available for analysis. In the last steps, we perform an interpretation of findings, which is an analysis of the results of the MEE to understand the level of complexity in China’s pilot carbon markets and to identify any trends or patterns in complexity levels across different time scales. In the final step of the framework, we should perform a comparison with the European market: here, we compare the complexity levels observed in the Chinese carbon market with those in the European market, as discussed in this paper. This comparison will help contextualize the findings and identify any differences or similarities between the two markets and deduce implications for policy and investment. Here, we use the findings of the MSE and MEE analysis to inform policy decisions and investment strategies related to the Chinese carbon market. For example, if the analysis reveals low complexity and market inefficiency, policymakers may consider implementing measures to improve market transparency and efficiency. Similarly, investors can use this information to make informed decisions about carbon trading in China. Hence, following these steps, we can effectively utilize the MSE evolution to gain insight into the complexity and dynamics of the Chinese carbon market, informing decision-making and policy formulation in the context of mitigating climate pollution.

## 3. Formulation of the General MED Framework

Nelson [36] derived the Schrödinger equation based on three assumptions—the background field hypothesis (Brownian motion), the requirements that Newton’s law gives the mean acceleration, and that the current velocity is a gradient. However, in a later work [37], Nelson avoided Newton’s law and required the diffusion process to be non-dissipative so that the expected energy is constant over time. However, this is a common feature of Bayesian or entropic inferences, where the goal is to update the prior probability to the posterior probability when new information becomes available. The new information could be in data (Bayesian inference) or constraints (entropic inference). In both cases, the two methods of inference are atemporal. It does not matter whether the posterior is obtained in the past or present; one obtains the same result. The ED framework differs from stochastic mechanics in ways that are delineated next. The ED formulation for a particular quantum system begins with defining the entropy functional subjected to relevant constraints of the system and defining the notion of entropic time. Relevant constraints are those that lead to the desired theory. Since our main concern is to derive quantum theory using ED, the relevant constraints are the phase and gauge constraints. Once the constraints are incorporated and the functional entropy is optimized, one obtains the transition probability, which is timeless. Interestingly, it is possible to introduce time in ED. Consider a particle that moves from the initial position *x* to the final position $x^{\prime }$. Generally, both positions are unknown. We deal with the joint probability $P(x,x^{\prime })$. Then, using the product rule of probability, we obtain the following:

(7) $$P\left(x,x^{\prime }\right)=P\left(x^{\prime }|x\right)P\left(x\right)\;,$$

where $P(x^{\prime }|x)$ is the probability of $x^{\prime }$ given *x*. Since *x* is also unknown, we marginalize over *x* to obtain the following:

(8) $$P\left(x^{\prime }\right)=\int dxP\left(x,x^{\prime }\right)=\int dxP\left(x^{\prime }|x\right)P\left(x\right)\;,$$

where P(x) is the probability of the particle being located at position *x*.

$P(x^{\prime })$ is the particle at position $x^{\prime }$. *x* occurs at an initial instant *t*, and $x^{\prime }$ occurs at a later instant $t^{\prime }$. Therefore, we finally set the probabilities in the following manner:

(9) $$P\left(x\right)=\rho \left(x,t\right)\;\;\mathrm{and}\;\;P\left(x^{\prime }\right)=\rho \left(x^{\prime },t^{\prime }\right)\;.$$

Time is introduced as a bookkeeping device in the ED formulation that keeps track of changes. The notion of time is further elaborated in Section 4, where the duration of time is also obtained.

The emergence of new information technologies has led to the rise of data science, which involves the analysis of large and complex datasets to make predictions about the evolution of systems. Data science has become essential for companies and organizations to gain insight into their customers and operations and make data-driven decisions. To perform a data analysis, one must first take a dataset and break it down into subsets. By analyzing the diversity of these subsets, one can determine the probabilities of realizing different outcomes. This approach is based on the principles of probability theory, which involves quantifying uncertainty and measuring the likelihood of other events. Using data analysis, one can use statistical mechanics to make predictions, which Boltzmann first introduced. This method is based on entropy, which measures the amount of disorder or randomness and the information in a system. By applying statistical mechanics to a dataset, one can determine the most likely outcomes and predict the system’s future evolution. We propose the following. Take a dataset and analyze it to decompose it into an arbitrary number of subsets. Then, using the diversity of those subsets, determine the probabilities for realizing the different subsets. Probabilities can be determined using statistical mechanics methods completely compatible with the presented MED methodology.

As we deal with the multiscale methodology, the additional ingredient is to sum over all scales representing the hierarchal system of particles or entities. The $Q(x^{\prime },s^{\prime }|x,s)$ is called the prior probability distribution, which we obtain from our dataset, as mentioned above. The unknown function is the transition probability $P(x^{\prime },s^{\prime }|x,s)$, which is analogous to the transition probability distribution for a single particle when it moves from position *x* to a neighboring point $x^{\prime }$, where $s,s^{\prime }$ are scaling indices or arbitrary variables of the dataset being considered. Our goal is to find the transition probability. But first, we have to find the prior, which we obtain from analyzing the information dataset under consideration.

The main idea of our approach is that in any system, with time, the entropy rises. The same happens in the case of the second law of thermodynamics, which deals with a complex system and many particles. So, we consider the dynamics of entropy in the MED methodology in the same way as it is done in the second law of thermodynamics. Therefore, our goal in this paper is to extend the application of ED [14] to a complex system that can be described using some complex dataset. The mathematical tools needed for a system of such directly non-interacting particles go beyond the usual statistics and calculus. Since a system of particles or a complex system can involve several constraints, one needs to adopt a multifaceted and multiscale approach to formulate the equations that describe the system accurately [38] (see the references therein). The proposed MED methodology has applications in both the natural and social sciences. For instance, the brain is a complex system of neurons, and in the same way, society is a complex system of communication networks. The universe itself is a complex system too, as it is comprised of planets, stars, and ultimately galaxies.

In this paper, we consider a complex system as an example of how to extend the ED method to the MED method, with the eventual goal of applying the MED method to the field of data science. As an illustration, we apply MED to complex systems under special constraints, and it results in a well-known form of the Generalized Schrödinger Equation (GSE). The set of equations obtained is identical to the nonlinear Schrödinger equations (NLSEs) that have been applied to various systems, including superconductivity. In other words, the equations derived under the MED approach describe the dynamics of nonlinear systems in physics consisting of plasmons, deformons, polarons, condensons, and optical or matter solitons. The dynamics of solitons are of great interest in describing the properties of new emerging fields of materials science, e.g., nonlinear optics, two-dimensional (2D) materials, cold and hot plasma physics, and crystal lattice dynamics. As an example, the matter solitons are considered non-relativistic quanta of matter waves and represent Bose–Einstein condensates (BECs) of atoms and electrons [39,40,41,42,43,44,44]. The first form of the NLSEs likely appeared in Landau’s phase transition theory, where he first introduced the cubic term in the Schrodinger equation that describes the order parameter [45]. Later, in 1950, this equation was applied by Ginzburg and Landau to superconductors, and the NLSE gained a new meaning, the famous Ginzburg–Landau equation [46]. Later, in the 1960s, the idea was applied to BECs, and the NLSE was named the Gross–Pitaevskii equation. On the other hand, an electron trapping by a crystal lattice was first described by Lev Landau in 1933 [39]. Solomon Pekar proposed the concept of the polaron in 1946 [47], which was further developed by Landau and Pekar in a 1948 paper [48]. This theory suggested that polarons, not free electrons, were the charge carriers in ionic crystals. Unlike quantum electrodynamics, the polaron theory is free from divergences, and the electron energy and mass remain finite. Today, research on polarons continues to expand into new areas of 2D materials, where new forms of NLSEs have been obtained [49]. In general, it would also be interesting to consider these new phenomena from the principle of MED and compare them with conventional approaches. In particular, for a description of nonlinear waves, the NSLE was originally derived by Zabusky and Kruskal [40], but using MED, one may include the physics of many non-equilibrium phenomena, dissipation, and scattering, and it can be used to describe the dynamics of solitons in 2D materials or many-body soliton physics.

## 4. Application of MED Methodology to a Complex System

As stated above, our eventual goal is to introduce the MED methodology to the field of data science. As an illustration, we apply it to describe the dynamics of quantum particles, namely solitons. However, a complex data science system may also have particles such as fractals, which are self-similar structures. Fractals are found in nature, in addition to being constructed experimentally and mathematically. Clouds, lightning, and coastlines are natural fractals, and the Sierpinski triangle is an example of geometrical fractals [50]. Moreover, geometrical objects are also fractals and can be found in Benoit B. Mandelbrot’s foundational book on fractals [51]. The creation of fractal solitons has been discussed in [52].

For a quantum statistical system, one must first specify the microstates, the prior probability distributions, and the constraints at the stage. Similarly, in a data science application, the prior probability distributions originated directly from the existing dataset which is the subject of the main complex system analysis. The most important part of this analysis is to find which constraints have been used in collecting the existing data. The correct evolution of entropy strongly depends on these constraints. In the next step, we must incorporate these limitations in the entropy function. With these taken into account, we arrive at a traditional generalized Boltzmann-like expression of entropy. To derive the linear Schrödinger equation (LSE), one considers *N* non-interacting particles living in a flat Euclidean space. It is assumed that particles have definite initial positions (and indefinite values of momenta) and yet-unknown values that are desired to be inferred. The different definite initial positions of the particles form a dataset. Such a dataset can be very large depending on how many initial positions for a single particle we will consider. The dataset can also be split into subsets associated with different scales, e.g., the fractal. Note that the microstates at each scale are different. The devised MED methodology is given below in the following steps.

### 4.1. MED Functional

At each scale, the particle is assumed to reside in a Euclidean space $X_{s}$ with metric $\delta_{ab}$, with a=1,2,3 for spatial coordinates. And for all particles at that scale, we have $X_{N_{s}}=X_{s}\times \ldots \times X_{s}$, which is $3N_{s}$-dimensional configuration space. The positions of the particles are given by $x_{i}^{a}\in X_{N_{s}}$, where the index $i=1,2,\ldots N_{s}$. We represent $x_{i}^{a}$ collectively by *x*. The multiscale entropic functional for a system can be written as (see a review on ED in [14]):

(10) $$S\left[P,Q\right]=-\sum_{s^{\prime }}\int d^{n}x^{\prime }P\left(x^{\prime },s^{\prime }|x,s\right)\log\frac{P(x^{\prime },s^{\prime }|x,s)}{Q(x^{\prime },s^{\prime }|x,s)}\;,$$

which is the extension of the functional entropy in [14]. As we are dealing with multiscale, the additional ingredient is to sum over all scales. Here, $Q(x^{\prime },s^{\prime }|x,s)$ is the prior probability distribution, and $P(x^{\prime },s^{\prime }|x,s)$ is the transition probability distribution as the particle moves from *x* to a neighboring point $x^{\prime }$, where $s,s^{\prime }$ are scaling indices. Our goal is to find the transition probability. But first, we have to determine the prior probability distribution. Any specific dataset can be obtained directly by classifying different snapshots. For the particular case of our many-particle system, as the ideal gas is to be determined prior, we will follow the original Boltzmann approach.

### 4.2. Prior Multiscale Probability Distribution Functional

The prior multiscale probability distribution functional $Q(x^{\prime },s^{\prime }|x,s)$ codifies the relation between *x* and $x^{\prime }$ before the information contained in constraints has been processed, where all particle positions are equally probable. In other words, we seek to find an invariant prior distribution under translation and rotation. It can be obtained by maximizing the following relative entropy:

(11) $$S\left(Q\right)=-\sum_{s^{\prime }}\int d^{n}x^{\prime }Q\left(\Delta x\right)\log\frac{Q(\Delta x)}{\mu (\Delta x)}\;,$$

where $\Delta x=x^{\prime }-x$ is relative to the uniform measure μ(Δx), subject to normalization and a constraint that concerns short steps,

(12) $$\sum_{s^{\prime }}\int d^{n}x^{\prime }Q\left(x^{\prime },s^{\prime }|x,s\right)\delta_{ab}\Delta x_{i}^{a}\Delta x_{i}^{b}=\langle \Delta \ell_{i}^{2}\rangle \;,\;\left(i=1,2,\ldots ,N_{s^{\prime }}\right)$$

where $\langle \Delta \ell_{i}^{2}\rangle$ are constants equal to the square of the average displacement between the points *x* and $x^{\prime }$. The index *i* indicates that $N_{s^{\prime }}$ constraints at each scale are rotational invariant, as given below.

(13) $$Q\left(x^{\prime },s^{\prime }|x,s\right)\propto \exp\left[-\frac{1}{2}\sum_{s^{\prime }}\sum_{i}^{N_{s^{\prime }}}\frac{1}{\sigma_{s^{\prime },i}^{2}}\delta_{ab}\Delta x_{i}^{a}\Delta x_{i}^{b}\right]$$

where $\sigma_{s^{\prime },i}^{2}$ is a Lagrange multiplier, which will be determined later (see Equation (20). To ensure small steps, this Lagrange multiplier must be very small. The right-hand side is the product of the Gaussian function, meaning that the short steps are independent. Equation (13) is a prior probability distribution that only takes into account the original positions of a particle before the actual constraints or information are incorporated, such as the influence of the EM field. It only describes motion in short steps as the particle moves from *x* to $x^{\prime }$. We want to write down a form of Equation (10) that works for describing the dynamics of particles and quasiparticles in solids, considering the case when the isotropic symmetry of the space is broken, e.g., by applying an external electric field. This symmetry breaking requires the inclusion of extra constraints listed below. In this way, the transition probability distribution $P(x^{\prime },s^{\prime }|x,s)$ will be determined. The isotropic symmetry breaking of the probability distribution can be achieved by introducing an external force per unit charge that depends on space. Consequently, it results in space-dependent symmetry breaking as well. In any complex system of data science, it is caused by the gradient of a scalar external potential. In this case of the complex system, it is the electric field generated by the gradient of electric “potential” $\phi_{s}\left(x^{a}\right)$ that satisfies the following constraint:

(14) $$\sum_{s^{\prime }}\int d^{n}x^{\prime }P\left(x^{\prime },s^{\prime }|x,s\right)\Delta x^{a}\frac{\partial \phi_{s}}{\partial x^{a}}=\kappa_{1,s}$$

This constraint is called the drift potential constraint in the context of a complex system. The $\kappa_{1,s}$ are constants related to equipotential lines. These equipotential lines are related to the cross-section perpendicular to the applied field.

The time-dependent symmetry breaking of the probability distribution can also be achieved by external force per unit charge that depends upon the time. This implies that a time-varying potential and a vector must generate the time-dependent component of the external force. For example, in the case of a complex system, the external electric field can also be generated by the rate change of the vector magnetic potential. This symmetry-breaking constraint can be imposed in the following form:

(15) $$\sum_{s^{\prime }}\int d^{n}x^{\prime }P\left(x^{\prime },s^{\prime }|x,s\right)\Delta x^{a}A_{a}=\kappa_{2}^{s}$$

where $A_{a}$ is the vector potential, which is a function of space and time, and the $\kappa_{2}^{s}$ are constants that represent the average displacement in the direction of the vector potential. Note that an arbitrary form of the vector potential can be selected, resulting in the meaningless form of symmetry breaking. Therefore, its gauge-invariant form must be selected. The gauge-invariant form of vector potential implies that it causes the symmetry breaking of the probability distribution functional simultaneously in time and space. This is why the symmetry breaking of the probability distributional functional in space due to the vector potential must be included, and it has been performed in the following way:

(16) $$\sum_{s^{\prime }}\int d^{n}x^{\prime }P\left(x^{\prime },s^{\prime }|x,s\right)\Delta x_{a}\epsilon^{abc}\frac{\partial A_{c}}{\partial x^{b}}=\kappa_{1,s}$$

Here, $\epsilon^{abc}$ is an anti-symmetric Levi–Civita symbol. This constraint is also called the drift potential constraint in the context of a complex system because of the drift of the vector potential. Therefore, in this case, $\kappa_{1,s}$ are the same constants related to equipotential lines.

### 4.3. Optimization of MED Functional

The maximized multiscale MED functional of the equation is subject to the constraints Equations (14) and (15). We obtain the following result for the transition probability by combining both constraints.

(17) $$P\left(x^{\prime },s^{\prime }|x,s\right)\propto \exp\left[-\frac{1}{2}\underset{s^{\prime }}{\sum }\overset{N_{s^{\prime }}}{\underset{i}{\sum }}\left(\frac{1}{\sigma_{s,i}^{2}}\delta_{ab}\Delta x_{i}^{a}\Delta x_{i}^{b}-\alpha_{s,i}^{\prime }\Delta x_{i}^{a}\frac{\partial \phi_{s}}{\partial x_{i}^{a}}+\beta_{s^{\prime },i}\Delta x_{i}^{a}A_{a}\right)\right]\;,$$

where $\sigma_{s,i}^{2}$, $\alpha_{s,i}^{\prime }$, and $\beta_{s^{\prime },i}$ are Lagrange multipliers, which will be expressed in the form of Planck’s constant *ℏ*, the speed of light, and the charge of an electron. For the sake of completeness, we should note that the gauge invariance of Equation (17) can be achieved and may be written in the following way:

(18) $$P\left(x^{\prime },s^{\prime }|x,s\right)\propto \exp\left[-\frac{1}{2}\underset{s^{\prime }}{\sum }\overset{N_{s^{\prime }}}{\underset{i}{\sum }}\left(\frac{1}{\sigma_{s,i}^{2}}\delta_{ab}\Delta x_{i}^{a}\Delta x_{i}^{b}-\alpha_{s,i}^{\prime }\Delta x_{i}^{a}\frac{\partial \phi_{s}}{\partial x_{i}^{a}}+\beta_{s^{\prime },i}\Delta x_{a}\epsilon^{abc}\frac{\partial A_{c}}{\partial x^{b}}\right)\right]\;.$$

In the MED formulation, we derive below the transition probability in the Gaussian form with time evolution. This formulation explicitly applies to Brownian motion in the general form of transition probability and entropic time. The entropic time is explained and derived next.

Any notion of time must involve motion and change [53]. In MED, motion or change is described by the transition probability given by (17). It is desired to obtain a small change. Large changes can be obtained by accumulating small or short steps. It should be noted that any notion of time must have (a) something one might identify as an instant, (b) a sense in which these instants can be ordered, and (c) a convenient concept of duration measuring the separation between instants [54]. In ED, an instant is defined by the information required to generate the next instant. The point *x* occurs at time *t*, and $x^{\prime }$ occurs at $t^{\prime }$. Therefore, probability distribution evolves according to

(19) $$\rho \left(x^{\prime },s^{\prime },t^{\prime }\right)=\sum_{s}\int dtdxP\left(x^{\prime },s^{\prime }|x,s\right)\rho \left(x,s,t\right).$$

We write $\rho \left(x,s,t\right)=\rho_{s}\left(x,t\right)$. Having introduced the notion of time, the next step is defining a time duration. Since our goal is to derive GSE, constructing a Newtonian interval independent of the position *x* and time *t* suffices. This can be achieved by the Lagrange multiplier $\sigma_{s,i}^{2}$ being constant such that

(20) $$\frac{1}{\sigma_{s,i}^{2}}=\frac{m_{i,s}}{\eta_{s}\Delta t}$$

where $m_{i,s}$ are the particle masses, and $\eta_{s}$ is a constant, which will be shown later to be *ℏ*. Furthermore

(21) $$M_{ab}=m_{s,i}\delta_{ab}\;,$$

where $M_{ab}$ is effective mass matrix. We have

(22) $$P\left(x^{\prime },s^{\prime }|x,s\right)\propto \exp\left[-\frac{1}{2\eta_{s}\Delta t}M_{ab}\left(\Delta x^{a}-\langle \Delta x^{a}\rangle \right)\left(\Delta x^{b}-\langle \Delta x^{b}\rangle \right)\right]\;.$$

Here,

(23) $$\Delta x^{a}=\langle \Delta x^{a}\rangle +\Delta w^{a}\;,$$

with

(24) $$\langle \Delta x^{a}\rangle =\eta_{s}\Delta tM^{ab}\left(\alpha_{s,i}^{\prime }\frac{\partial \phi_{s}}{\partial x^{b}}-\beta_{s}A_{b}\right)\;,$$

(25) $$\langle \Delta w^{a}\rangle =0\;\;\;\mathrm{and}\;\;\langle \Delta w^{a}\Delta w^{b}\rangle =\eta_{s}\Delta tM^{ab}\;.$$

This is Brownian motion because of the drift $\langle \Delta x^{a}\rangle ∼O\left(\Delta t\right)$ and the fluctuation $\Delta w^{a}∼O\left(\Delta t^{1/2}\right)$. The trajectory is continuous but not differentiable.

### 4.4. Dynamic Representation of MED Functional

The probability $\rho_{s}\left(x,t\right)$ evolves according to the Fokker–Planck (FP) equation given below.

(26) $$\begin{array}{c} \frac{\partial \rho_{s}}{\partial t}=-\partial_{a}\left(v_{s}^{a}\rho_{s}\right)\;. \end{array}$$

Note that *s* is the scaling index, and a=1,2,3 are spatial indices. A summation over repeated indices should be understood. Here, $v^{a}$ is the current velocity given by

(27) $$v_{s}^{a}=M^{ab}\left(\alpha_{s,i}^{\prime }\partial_{a}\Phi_{s}-\beta_{s}A_{a}\right)\;$$

where $\Phi_{s}=\alpha_{s,i}^{\prime }\eta_{s}\phi_{s}-\eta_{s}\log\rho_{s}^{1/2}$ So far, we only have one dynamical variable, $\rho_{s}$, which evolves according to Fokker–Planck (FP) equation. To derive the Schrödinger equation, we need two dynamical variables, the probability $\rho_{s}$ and phase $\Phi_{s}$. To promote $\Phi_{s}$ to a dynamical variable, we need another constraint $H=H(\rho_{s},\Phi_{s})$, where *H* is an energy functional. By requiring that the energy is conserved, we also obtain the second dynamical variable. The functional $H(\rho_{s},\Phi_{s})$ can be constructed by writing the FP equation as

(28) $$\frac{\partial \rho_{s}}{\partial t}=\frac{\delta H}{\delta \Phi_{s}}\;.$$

It can easily be checked that the appropriate energy function is given by

(29) $$H\left(\rho_{s},\Phi_{s}\right)=\sum_{s}\int dx\rho_{s}\left(\frac{1}{2}M^{ab}\left(\partial_{a}\Phi_{s}-\beta_{s}A_{a}\right)\left(\partial_{b}\Phi_{s}-\beta_{s}A_{b}\right)+V_{s}\left(x\right)\right)+\underset{ss^{\prime }}{\sum }g_{ss^{\prime }}F\left(\rho_{s},\rho_{s^{\prime }}\right)\;,$$

where V(x) is a scalar potential, $F(\rho_{s},\rho_{s^{\prime }})$ is an integration to be determined below, and $g_{ss^{\prime }}$ is the complexity coefficient. This term leads to the nonlinear Schrödinger equation. We have

(30) $$\frac{\delta H}{\delta \rho_{s}}=\frac{1}{2}M^{ab}\left(\partial_{a}\Phi_{s}-\beta_{s}A_{a}\right)\left(\partial_{b}\Phi_{s}-\beta_{s}A_{b}\right)+V_{s}\left(x\right)+\underset{s^{\prime }}{\sum }g_{ss^{\prime }}\frac{\delta F(\rho_{s},\rho_{s^{\prime }})}{\delta \rho_{s}}$$

Taking total time derivative of Equation (29) and require it to be conserved and also incorporate Equation (28),

(31) $$\frac{dH}{dt}=\sum_{s}\int dx\left[\frac{\delta H}{\delta \Phi_{s}}\partial_{t}\Phi_{s}+\frac{\delta H}{\delta \rho_{s}}\partial_{t}\rho_{s}\right]=\sum_{s}\int dx\left[\partial_{t}\Phi_{s}+\frac{\delta H}{\delta \rho_{s}}\right]\partial_{t}\rho_{s}=0$$

It holds for all $\partial_{t}\rho_{s}$, which means that

(32) $$\frac{\partial \Phi_{s}}{\partial t}=-\frac{\delta H}{\partial \rho_{s}}\;.$$

We obtain

(33) $$\frac{\partial \Phi_{s}}{\partial t}=-\frac{1}{2}M^{ab}\left(\partial_{a}\Phi_{s}-\beta_{s}A_{a}\right)\left(\partial_{b}\Phi_{s}-\beta_{s}A_{b}\right)-V_{s}-\underset{s^{\prime }}{\sum }g_{ss^{\prime }}\frac{\delta F(\rho_{s},\rho_{s^{\prime }})}{\delta \rho_{s}}\;.$$

This is the quantum Hamilton–Jacobi equation. Equations (26) and (33) can be combined using

(34) $$\psi_{s}=\rho_{s}^{1/2}\exp\left[ik\Phi_{s}/\eta_{s}\right]\;.$$

The result is

(35) $$\begin{array}{ccc} \frac{i\eta_{s}}{k}\frac{\partial \psi_{s}}{\partial t} & = & \frac{\eta_{s}^{2}}{2k^{2}}M^{ab}\left(i\partial_{a}-\beta_{s}A_{a}\right)\left(i\partial_{b}-\beta_{s}A_{a}\right)\psi_{s}+V_{s}\psi_{s}+\frac{\eta_{s}^{2}}{2k^{2}}\frac{M^{ab}\partial_{a}\partial_{b}\sqrt{\rho_{s}}}{\sqrt{\rho_{s}}}\psi_{s}\\ & + & \underset{s^{\prime }}{\sum }g_{ss^{\prime }}\frac{\delta F(\rho_{s},\rho_{s^{\prime }})}{\delta \rho_{s}}\psi_{s}\;, \end{array}$$

### 4.5. Gauge Invariance of MED Derived Relations

The physical meaning of the $\psi_{s}$ in Equation (34) is that it represents the wavefunction of the particle of the generalized Schrödinger equation. Its modulus is the probability of finding the particle in space and time. In most general situations, for example, in data science, $\psi_{s}$ will be the parameter controlling the transition probabilities. Note that Equation (35) is invariant under the gauge transformation given below [55].

(36) $$\psi_{s}\to \psi_{s}^{\prime }=e^{i\beta \chi (x,t)}\psi_{s}\;\;\mathrm{and}\;\;A_{a}\to A_{a}^{\prime }=A_{a}+\partial_{a}\chi .$$

In Equation (35), the third term on the right is called the quantum potential. Normally, this term is present in the Hamilton–Jacobi Equation (33). By combining this equation with the Fokker–Planck equation, Equation (26), one obtains a linear Schrödinger equation (LSE) that obeys the superposition principle. The quantum potential is implicit in $F(\rho_{s},\rho_{s^{\prime }})$. Since we have freedom in the choice of *F*, we choose it such that

(37) $$\underset{s^{\prime }}{\sum }g_{ss^{\prime }}\frac{\delta F(\rho_{s},\rho_{s^{\prime }})}{\delta \rho_{s}}+\frac{\eta_{s}^{2}}{2k^{2}}\frac{M^{ab}\partial_{a}\partial_{b}\sqrt{\rho_{s}}}{\sqrt{\rho_{s}}}=\underset{s^{\prime }}{\sum }g_{ss^{\prime }}f\left(\rho_{s^{\prime }}\right)\;.$$

Note that the function *f* on the right is only a function of one variable. If the goal is to obtain an LSE, one can set f=0. However, we are interested in nonzero *f* for the reasons given below.

(38) $$i\hbar \frac{\partial \psi_{s}}{\partial t}=\frac{\hbar^{2}}{2}M^{ab}\left(i\partial_{a}-\frac{e}{\hbar c}A_{a}\right)\left(i\partial_{b}-\frac{e}{\hbar c}A_{a}\right)\psi_{s}+V_{s}\psi_{s}+\underset{s^{\prime }}{\sum }g_{ss^{\prime }}f\left(\rho_{s^{\prime }}\right)\psi_{s}\;.$$

Here, we used $\eta_{s}/k=\hbar$ and $\beta_{s}=e/\hbar c$, where *e* is the charge of an electron, and *c* is the speed of light. Equation (38) is the sought Generalized Nonlinear Schrödinger Equation (GNSE), which takes into account the interaction of the electromagnetic field with matter waves. The last term indicates nonlinearity. A similar last term is also reported in [52]. But here, we naturally derived the general NLSE using entropic dynamics. For solitons, we can take $f\left(\rho_{s^{\prime }}\right)=\rho_{s^{\prime }}={\left|\psi_{s^{\prime }}\right|}^{2}$. So,

(39) $$i\hbar \frac{\partial \psi_{s}}{\partial t}=\frac{\hbar^{2}}{2}M^{ab}\left(i\partial_{a}-\frac{e}{\hbar c}A_{a}\right)\left(i\partial_{b}-\frac{e}{\hbar c}A_{b}\right)\psi_{s}+V_{s}\psi_{s}+\underset{s^{\prime }}{\sum }g_{ss^{\prime }}{\left|\psi_{s^{\prime }}\right|}^{2}\psi_{s}\;.$$

The existence of the nonlinear terms [56,57,58] and, in particular, the cubic term [59] is characteristic of the solitons and other nonlinear waves (see the seminal paper by Zakharov [60] about the nonlinear stability of periodic waves in deep water). The existence of such waves and stable solitons depends on the boundary conditions, and their dynamical stability depends on the spatial dimension of the system [59,61]. In addition to solitons, there is a large variety of nonlinear phenomena, including shape waves [58], periodic waves in deep and shallow water [60], plasma cavitons [61], Urbach and Lifshiz density of state tails [62], the collapse of the plasmon, Langmuir waves [63], and many other related phenomena [64]. It is very interesting if the NSE equations describing these or associated phenomena can be obtained with the principle of the maximum entropy and entropic dynamics described above.

## 5. Reduction of GNSE to a Few Relevant Representations

One can note that Equation (39) is a complex system of equations. It has some general form, which covers numerous physical phenomena. It may have scalar or vector (tensor) forms [63,65]. Below, we will discuss those forms of the NLSEs that found direct applications in different areas of physics, and it covers not only solitons but other quasiparticles, too [65,66], including the phenomena such as self-trapping and polarons [67] as well as plasma caviton formation [68,69]. Here, we consider the simplest example where two solitons coupled to each other may be created [70]. In one case, we obtain the vector nonlinear Schrödinger equation (VNSE). In the other case, the scalar nonlinear Schrödinger equation (SNSE) is obtained. The difference between the VNSE and SNSE is that the former involves coupled solitons and decoupled solitons and many different physical phenomena.

### 5.1. Scalar Form of GSE for Decoupled Solitons

The solitons usually exist in a one-dimensional chain or system with reduced dimensions [59]. The illustrative example is Davydov solitons [70], created in protein chains. They are associated with electron self-trapping or localization of protein Amide-I (or CO stretching) vibrational energy. Such localization, as well as electron self-trapping, arises through the interaction of the Amide-I mode with lattice distortion and plays an essential role in the charge transport vital for all biological systems. Our starting point is the system (39). For illustration, the EM field is set to zero ($\vec{A}=0$). The SNSE can be obtained by setting

(40) $$g_{ss^{\prime }}=0,\;\;\mathrm{when}\;\;s\neq s^{\prime }$$

where $g_{11}$ and $g_{22}$ survive. Further set $\eta_{s}/k=\hbar$. We obtain two decoupled SNSEs as follows:

(41) $$i\hbar \frac{\partial \psi_{1}}{\partial t}=-\frac{\hbar^{2}}{2m}\nabla^{2}\psi_{1}+V_{1}\psi_{1}+g_{11}{\left|\psi_{1}\right|}^{2}\psi_{1}\;.$$

(42) $$i\hbar \frac{\partial \psi_{2}}{\partial t}=-\frac{\hbar^{2}}{2m}\nabla^{2}\psi_{2}+V_{2}\psi_{2}+g_{22}{\left|\psi_{2}\right|}^{2}\psi_{2}\;,$$

which is the desired system of two decoupled solitons. The last two equations are the Gross–Pitaevski equation (GPE) [52,71]. In Bose–Einstein condensates (BECs), g<0 is referred to as the bright solitons, and g>0 is called the dark solitons [71].

### 5.2. A Vector Form of the GSE for Coupled Solitons

For illustration, we again recall the system (39) with $\vec{A}=0$. Set $g_{ss^{\prime }}$ such that

(43) $$g_{ss^{\prime }}=0,\;\;\mathrm{when}\;\;s=s^{\prime }\;,$$

where $s,s^{\prime }=1,2$. Equation (39) simplifies to

(44) $$i\hbar \frac{\partial \psi_{1}}{\partial t}=-\frac{\hbar^{2}}{2m}\nabla^{2}\psi_{1}+V_{1}\psi_{1}+g_{12}{\left|\psi_{2}\right|}^{2}\psi_{1}\;,$$

(45) $$i\hbar \frac{\partial \psi_{2}}{\partial t}=-\frac{\hbar^{2}}{2m}\nabla^{2}\psi_{2}+V_{2}\psi_{2}+g_{21}{\left|\psi_{1}\right|}^{2}\psi_{2}\;,$$

which is the desired system of two solitons. Generally, we have

(46) $$i\hbar \frac{\partial \psi_{1}}{\partial t}=-\frac{\hbar^{2}}{2m}\nabla^{2}\psi_{1}+V_{1}\psi_{1}+\hbar g_{11}|\psi_{1}{|}^{2}\psi_{1}+g_{12}{\left|\psi_{2}\right|}^{2}\psi_{1}\;,$$

(47) $$i\hbar \frac{\partial \psi_{2}}{\partial t}=-\frac{\hbar^{2}}{2m}\nabla^{2}\psi_{2}+V_{2}\psi_{2}+\hbar g_{21}|\psi_{1}{|}^{2}\psi_{2}+g_{22}{\left|\psi_{2}\right|}^{2}\psi_{2}\;.$$

### 5.3. The Interaction of Electromagnetic Fields with Coupled and Decoupled Solitons

We can also write the full equation for solitons with EM fields. Recall Equation (39)

(48) $$i\hbar \frac{\partial \psi_{s}}{\partial t}=\frac{\hbar^{2}}{2}M^{ab}\left(i\partial_{a}-\frac{e}{\hbar c}A_{a}\right)\left(i\partial_{b}-\frac{e}{\hbar c}A_{a}\right)\psi_{s}+V_{s}\psi_{s}+\underset{s^{\prime }}{\sum }g_{ss^{\prime }}{\left|\psi_{s^{\prime }}\right|}^{2}\psi_{s}\;.$$

where $M^{ab}=\delta^{ab}/m_{i}$ is the the inverse of mass matrix. For coupled solitons, we have

(49) $$i\hbar \frac{\partial \psi_{1}}{\partial t}=\frac{\hbar^{2}}{2m}\delta^{ab}\left(i\partial_{a}-\frac{e}{\hbar c}A_{a}\right)\left(i\partial_{b}-\frac{e}{\hbar c}A_{a}\right)\psi_{1}+V_{1}\psi_{1}+g_{12}{\left|\psi_{2}\right|}^{2}\psi_{1}\;.$$

(50) $$i\hbar \frac{\partial \psi_{2}}{\partial t}=\frac{\hbar^{2}}{2m}\delta^{ab}\left(i\partial_{a}-\frac{e}{\hbar c}A_{a}\right)\left(i\partial_{b}-\frac{e}{\hbar c}A_{a}\right)\psi_{2}+V_{2}\psi_{2}+g_{21}{\left|\psi_{1}\right|}^{2}\psi_{2}\;.$$

Similarly, for decoupled solitons, we have

(51) $$i\hbar \frac{\partial \psi_{1}}{\partial t}=\frac{\hbar^{2}}{2m}\delta^{ab}\left(i\partial_{a}-\frac{e}{\hbar c}A_{a}\right)\left(i\partial_{b}-\frac{e}{\hbar c}A_{a}\right)\psi_{1}+V_{1}\psi_{1}+g_{11}{\left|\psi_{1}\right|}^{2}\psi_{1}\;.$$

(52) $$i\hbar \frac{\partial \psi_{2}}{\partial t}=\frac{\hbar^{2}}{2m}\delta^{ab}\left(i\partial_{a}-\frac{e}{\hbar c}A_{a}\right)\left(i\partial_{b}-\frac{e}{\hbar c}A_{a}\right)\psi_{2}+V_{2}\psi_{2}+g_{22}{\left|\psi_{2}\right|}^{2}\psi_{2}\;.$$

In summary, Equations (41)–(52) are special cases of Equation (39). Generally, the scaling indices $s,s^{\prime }$ may vary as 1,2,…n that describe a system of *n* coupled equations or quasiparticles. It is also worth noting that the coupling enters through the complex coefficient $g_{ss^{\prime }}$. If it is set to zero, Equation (39) reduces to the linear SE that obeys the usual superposition principle for the particle system *n*.

## 6. Temperature Dependent Dynamics of Complex Systems

It was shown in Section 4 that the dynamics of a complex system evolve according to the GNSE. The GNSE does not address the description of the properties of such a complex system in the form of temperature and pressure. This can be achieved by connecting the entities of a complex system with temperature and pressure using the Gibbs–Duhem equation, which relates the chemical potential μ to entropy and molar volume. It is given in [72], and it can be noticed from there that the chemical potential (μ) of a complex system can be expressed in the following form:

(53) μ=μ(T,P)

In differential form, we have

(54) $$\begin{array}{c} d\mu =-SdT+VdP \end{array}$$

which is the Gibbs–Duhem equation. Here,

(55) $$S=-{\left(\frac{\partial \mu }{\partial T}\right)}_{P}\;\;\;\mathrm{and}\;\;\;V={\left(\frac{\partial \mu }{\partial P}\right)}_{T}$$

In our case, the entropy is given by (10), which can be computed by plugging in Equations (13) and (22) and integrate. The result is

(56) $$S=\frac{m}{2\hbar \Delta t}\sum_{s}{\left(\frac{3\pi^{1/2}\hbar^{3/2}{\left(\Delta t\right)}^{3/2}}{m^{3/2}}\right)}^{3N_{s}}=\mathrm{constant}=C_{1}$$

One observes that the MED entropy is constant. This makes sense because the goal of MED is to maximize entropy, subject to constraints. Since *S* is a constant, this gives the chemical potential given by Equation (55),

(57) $$\mu =-C_{1}T+\alpha \left(P\right)$$

where α(P) is another constant that depends on pressure. Equation (57) can be considered the solution of the Gibbs–Duhem equation in the MED formalism. It should be noted that data science techniques can be applied to quantities such as those given in the above equation to determine the temperature- and pressure-dependent dynamics of complex systems.

## 7. Discussion

The burgeoning field of data science has been significantly influenced by insights drawn from statistical mechanics and physics, leading to the development of numerous innovative methods for analyzing complex datasets. Central to this integration is the foundational concept of statistical mechanics entropy, employed in data science to quantify uncertainty and information content. In particular, Shannon entropy, cross-entropy, and Kullback–Leibler divergence have found valuable applications in information theory and machine learning [73]. Furthermore, principles and models originating from statistical mechanics, such as the Ising model for magnetic systems and spin glasses for disordered magnetic systems, have been seamlessly adapted to address challenges in data science, particularly in the realms of clustering, pattern recognition, and optimization problem solving [74,75,76]. Utilizing Monte Carlo methods, renowned in statistical physics for numerical simulations, has proven instrumental in data science in addressing optimization, integration, and sampling problems. Markov chain Monte Carlo (MCMC) techniques, a subset of these methods, excel in exploring high-dimensional spaces and estimating probabilistic distributions [77,78,79]. Percolation theory, initially conceived in physics to study connected clusters in random networks, has practical applications in data science, especially in analyzing connectivity and network structures. This is particularly relevant in fields such as social network analysis and transportation network modeling [80]. Moreover, adapting renormalization group methods, essential to studying phase transitions in physics, has facilitated feature extraction and dimensionality reduction in data science. These methods play a vital role in identifying relevant scales and simplifying complex datasets [81,82]. The infusion of concepts from quantum mechanics has spurred the development of quantum computing algorithms, showcasing the potential to improve the efficiency of solving specific problems in data science. Quantum machine learning algorithms actively explore using quantum properties for improved computational performance [83]. Additionally, techniques derived from dynamical systems and chaos theory, commonly applied in physics, have found applications in data science for time series analysis and forecasting. Nonlinear dynamics methods are particularly adept at revealing hidden patterns and structures in temporal data [84,85,86]. The extension of principles from statistical mechanics to model learning processes, known as the statistical mechanics of learning, offers valuable insights into the generalization capabilities of machine learning models. This understanding helps to understand these models’ behavior regarding the data’s size and complexity [87]. Incorporating these concepts from statistical mechanics and physics into data science consistently enriches the field with powerful tools, empowering researchers to explore and understand the intricate underlying principles governing complex systems and datasets. The presented work can potentially impact the solution of more complex, challenging data science systems in various ways, including those from physics, cosmology, and statistical mechanics. Their short description is discussed next, along with results from the work presented in the manuscript and the literature.

In the 1950s, Ginsburg and Landau proposed the Ginsburg–Landau (GL) functional for free energy to describe the superconducting state in solids [46]. The GL functional was developed to introduce a nonlinear term in the Schrödinger equation (SE) to describe the superconductivity property of conductors by presenting the conduction electrons as superfluids. The minimization of this GL functional gives rise to the nonlinear Schrödinger equation (NLSE). The NLSE describes a new state of quasiparticles, the superconducting condensate, similar to Bose–Einstein condensation [40]. Gross and Pitaevskii derived the NLSE by applying the minimum energy principle to the free energy of condensed paired electrons and atoms in the Bose–Einstein condensation state. The use of NLSE enabled the description of the quantum dynamics of other systems in the form of solitary matter waves, solitons, or polarons. It can also be applied to a system consisting of coupled solitons containing multi-solitons or having optically interacting solitons.

The NLSE has been widely used in many fields of physics, including condensed matter physics, nonlinear optics, and fluid dynamics. Solitons, which are localized wave packets that maintain their shape during propagation, have been described by the NLSE. These solitons can exist in many systems, such as optical fibers, plasmas, and superfluids. The maximization of the MED functional not only resulted in an extension of GPE but also provided a natural way to include other interactions, such as the interaction of electromagnetic fields with quasiparticles in solids. Further, it provides the tools to deal with the scalar’s dynamics and vector solitons in decoupled and coupled forms. However, it should be noted that the GPE deals with scalar solitons. The implications of the GNSE may be quite far-reaching, in our opinion. Its application to 2D materials may lead to opportunities for discovering the quantized energies of solitons at the defect sites of those materials. Such quantized states may turn out to be suitable for future applications in electronics, including quantum computing.

In addition, the presented MED methodology is envisioned as complementary to statistical methods currently applied to existing datasets to forecast the future behavior of complex systems. The entire temporal evolution is intricately linked with a complex dataset and is delineated by a system of interconnected nonlinear Schrödinger equations. This methodology draws inspiration from entropy evolution, analogous to the second law of thermodynamics, considered one of the most elegant laws in physics. Naturally, any complex system tends to evolve towards equilibrium and stability. However, over short durations, the system might become trapped by false minima, leading to potential discrepancies in the predictions of the developed theory. Hence, in the short term, the theory’s predictions may falter. Nonetheless, machine learning can yield favorable outcomes in such instances. Conversely, machine learning predictions may prove less reliable in the long term, while the approach grounded in the second law remains robust. This resilience is attributed to its foundation in the core principles of statistical mechanics.

In our research effort, we provide the underlying principles that can be used to perform advanced statistical analyses on existing datasets to gain insight into and forecast the future behavior of complex systems. This predictive modeling will comprehensively explore the entire temporal evolution intricately linked with a complex dataset. The dynamic behavior of the system is delineated by a set of interconnected nonlinear Schrödinger equations, where time may be a complex variable reflecting the intricate interplay of various factors such as stochastic and diffusion processes [88,89,90,91,92]. The innovative methodology draws inspiration from entropy evolution, a concept analogous to the revered second law of thermodynamics, considered one of the most elegant principles in physics. According to the second law, any complex system naturally tends to evolve towards a state of equilibrium and stability. However, over shorter durations, the system may encounter challenges and become ensnared by false minima. Such instances can lead to discrepancies in the predictions of our developed theory, particularly in the short term, where the theory’s predictive accuracy may falter. Despite these short-term challenges, we recognize the potential of machine learning algorithms to yield favorable outcomes in instances where our theoretical predictions may be less reliable. Conversely, in the long term, our approach grounded in the second law remains robust due to its foundation in the core principles of statistical mechanics. Minimization of free energy, which encompasses both internal energy and entropy, provides a more comprehensive perspective on the thermodynamic behavior of systems [88,89,90,91,92]. While our approach draws inspiration from the elegance of the second law of thermodynamics and its connection to entropy evolution, we acknowledge the importance of considering the broader context of free-energy minimization, especially in environments where temperature plays a significant role. It should be noted that new methods are emerging to study economic uncertainty using physics-based stochastic differential equations [93]. Future developments in our research will incorporate these insights to provide a more nuanced understanding of complex systems and their evolution. In a recent article based on an analysis of a huge database, the relationship between DNA methylation and mutability, specifically how methylation can affect the emergence of new genetic variations in eukaryotes (organisms with cells containing a nucleus), has been identified [94]. Further analysis of somatic mutation data, particularly from cancers where specific repair pathways are compromised, is necessary to understand the underlying mechanisms of this process and the involvement of particular DNA repair pathways as well as how the impact of methylation on mutability extends beyond the methylated cytosine itself. DNA methylation, a common epigenetic modification in eukaryotes, can affect genetic variation in ways that are not fully understood and imply that the precise mechanisms involved are complex and require further investigation [94]. Their findings suggest that methylation significantly impacts the emergence of new genetic variants in eukaryotes, which may have important implications for understanding genetic diversity and disease. We hope that applying the proposed MED methodology to huge databases can further shed light on their evolutionary mechanisms [94]. In addition, all three fields are concerned with extracting meaningful information from noisy and complex datasets. Statistical physics seeks to identify and understand the underlying patterns and structures that govern the behavior of physical systems. Information theory seeks to extract and transmit useful information from noisy or uncertain datasets. Data science seeks to extract insights and knowledge from large, complex datasets.

Our research paves the way for applying sophisticated statistical analyses of existing datasets to gain insight and forecast the future behavior of complex systems. This involves a comprehensive exploration of temporal evolution intricately linked with a complex dataset, represented by a system of interconnected nonlinear Schrödinger equations [88,89]. Several factors are present in this formulation. First, the two crucial aspects of our entropy evolution method, entropy maximization and Gibbs energy minimization, are discussed in recent references [88,89]. Entropy maximization determines the equilibrium state of mixtures with specified total internal energy and total volume, while Gibbs energy minimization corresponds to mixtures with a specified pressure and temperature. Our findings show promise in addressing both problems, and the particle swarm optimization method performs well, particularly in Gibbs energy minimization [89]. Second, the computational scheme for temporal evolution under MED formulation outlines a method to depict the temporal evolution of thermodynamic functions in stochastic non-equilibrium processes of arbitrary classical systems. This scheme is particularly suited for representing the dynamic behaviors of non-equilibrium molecular systems, such as the conformational changes observed in protein folding and ligand docking [90]. Third, the diffusion Monte Carlo method can relax the dynamics determined under MED formulation. For instance, Tanaka’s use of the diffusion Monte Carlo method to simulate relaxation dynamics in classical systems, considering various model potentials, reveals intriguing results. Depending on initial conditions and potential landscapes, either an increase in entropy dominates the relaxation dynamics toward equilibrium or the dynamics are driven by the decrease in enthalpy, leading to a decrease in entropy associated with spatial localization [91,92]. Fourth, the role of the MED methodology in data science can be established by noting that MED methodology is applicable at the primary level. In contrast, data science applies to secondary-level quantities of complex systems. The multiscale entropy dynamics (MED) methodology emerges as a powerful tool in data science, offering a unique lens to analyze the intricate interplay of physical agents in dynamic systems [91]. Applying MED to data science involves viewing physical agents as entities driven by causal entropic forces, akin to a Darwinian perspective where they compete to consume future histories [91]. Fifth, the presented MED methodology also applies to biology. The free energy principle (FEP) serves as a theoretical framework, emphasizing the minimization of free energy in living systems [92]. MED, like FEP, introduces the concept of Markov blankets and emphasizes the relationship between action and perception in biological systems [91]. These frameworks provide insights into neural processes, learning, and decision-making, offering a unifying perspective on the behavior of living systems.

## 8. Conclusions

The MED formulation nicely describes the microscale dynamics of complex systems and paves the way for the subsequent application of data science tools. For example, it was shown that using the MED formulation, the dynamics of a complex system of particles can be expressed as a GNSE. Furthermore, the GNSE is adept at describing the motion of single particles or many noninteracting quasiparticles, such as polarons and solitons. There is an aspiration to apply the MED methodology to elucidate the dynamics of polarons and solitons in two-dimensional materials. Furthermore, the particles or quasiparticle system can be treated as a quantum gas. Therefore, applying the Gibbs–Duhem formulation to quantum gas can allow the MED-determined quantities to be related to temperature and pressure. Additionally, the intertwining of machine learning and artificial intelligence techniques has further solidified the connection between complex systems and data science. These techniques, extensively employed in data science, rely on statistical and probabilistic models that resemble those employed in statistical physics and information theory. For instance, deep learning models are constructed upon neural networks akin to those used in statistical physics to model the behavior of physical systems. This convergence of methodologies holds promise for advancing our understanding in diverse scientific domains.

In summary, the multiscale entropy (MSE) formulation serves as a powerful tool for characterizing the steady-state complexity of a diverse array of systems. However, the multiscale entropy evolution (MEE) framework and multiscale entropy dynamics (MED) methodology enable the investigation of the dynamic nature of complexity within these systems. This is how our research contributes to developing accurate and efficient methods for performing phase-equilibrium computations and understanding the nature of data in complex systems. The MED methodology showcases its versatility in various scientific and industrial domains, providing a unifying perspective on the behavior of living systems and complex processes. The statistical physics, information theory, and data science fields are connected by using similar mathematical and statistical tools to model and analyze complex systems. These connections have become stronger with machine learning and artificial intelligence techniques heavily based on the probabilistic and statistical models developed in these fields. The MED method can be adopted to improve the accuracy of data analysis, which involves optimizing the subset selection process to minimize the error in predicting the system’s evolution. We expect this method will be effective in various applications, including finance, healthcare, and social media analysis.

## Data Availability

No data were used in this study because the manuscript is based on a theoretical or mathematical work.

## References

[1] Jaynes E.T. (1957). Information theory and statistical mechanics. II. Phys. Rev..

[2] Sega M., Faccioli P., Pederiva F., Garberoglio G., Orland H. (2007). Quantitative protein dynamics from dominant folding pathways. Phys. Rev. Lett..

[3] Wehrl A. (1978). General properties of entropy. Rev. Mod. Phys..

[4] Pressé S., Ghosh K., Lee J., Dill K.A. (2013). Principles of maximum entropy and maximum caliber in statistical physics. Rev. Mod. Phys..

[5] Johnson D.T., Caticha A. (2012). Entropic dynamics and the quantum measurement problem. AIP Conf. Proc..

[6] Vanslette K., Caticha A. (2017). Quantum measurement and weak values in entropic dynamics. AIP Conf. Proc..

[7] Nawaz S., Caticha A. (2012). Momentum and uncertainty relations in the entropic approach to quantum theory. AIP Conf. Proc..

[8] Nawaz S., Abedi M., Caticha A. (2016). Entropic dynamics on curved spaces. AIP Conf. Proc..

[9] Ipek S., Caticha A. (2015). Entropic quantization of scalar fields. AIP Conf. Proc..

[10] Ipek S., Abedi M., Caticha A. (2019). Entropic dynamics: Reconstructing quantum field theory in curved space-time. Class. Quantum Gravity.

[11] Ipek S., Caticha A. (2020). The Entropic Dynamics of Quantum Scalar Fields Coupled to Gravity. Symmetry.

[12] Bai L., Cui L., Zhang Z., Xu L., Wang Y., Hancock E.R. (2020). Entropic dynamic time warping kernels for co-evolving financial time series analysis. IEEE Trans. Neural Netw. Learn. Syst..

[13] Caticha A. (2011). Entropic dynamics, time and quantum theory. J. Phys. A Math. Theor..

[14] Caticha A. (2019). The Entropic Dynamics Approach to Quantum Mechanics. Entropy.

[15] Bohm D., Hiley B.J. (2006). The Undivided Universe: An Ontological Interpretation of Quantum Theory.

[16] Kusmartsev F. (2011). Statistical mechanics of economics I. Phys. Lett. A.

[17] Bar-Yam Y. (2004). Multiscale complexity/entropy. Adv. Complex Syst..

[18] Bar-Yam Y. (2004). Multiscale variety in complex systems. Complexity.

[19] Bar-Yam Y. (2004). A mathematical theory of strong emergence using multiscale variety. Complexity.

[20] Humeau-Heurtier A. (2020). Multiscale entropy approaches and their applications. Entropy.

[21] Bar-Yam Y. (2006). Improving the effectiveness of health care and public health: A multiscale complex systems analysis. Am. J. Public Health.

[22] McDonough I.M., Letang S.K., Erwin H.B., Kana R.K. (2019). Evidence for maintained post-encoding memory consolidation across the adult lifespan revealed by network complexity. Entropy.

[23] Menon S.S., Krishnamurthy K. (2019). A study of brain neuronal and functional complexities estimated using multiscale entropy in healthy young adults. Entropy.

[24] De Wel O., Lavanga M., Caicedo A., Jansen K., Naulaers G., Van Huffel S. (2019). Decomposition of a multiscale entropy tensor for sleep stage identification in preterm infants. Entropy.

[25] Jelinek H.F., Cornforth D.J., Tarvainen M.P., Khalaf K. (2019). Investigation of linear and nonlinear properties of a heartbeat time series using multiscale Rényi entropy. Entropy.

[26] El-Yaagoubi M., Goya-Esteban R., Jabrane Y., Muñoz-Romero S., García-Alberola A., Rojo-Álvarez J.L. (2019). On the robustness of multiscale indices for long-term monitoring in cardiac signals. Entropy.

[27] Lin T.K., Chien Y.H. (2019). Performance evaluation of an entropy-based structural health monitoring system utilizing composite multiscale cross-sample entropy. Entropy.

[28] Ge M., Lv Y., Zhang Y., Yi C., Ma Y. (2019). An effective bearing fault diagnosis technique via local robust principal component analysis and multi-scale permutation entropy. Entropy.

[29] Perpetuini D., Chiarelli A.M., Cardone D., Filippini C., Bucco R., Zito M., Merla A. (2019). Complexity of frontal cortex fNIRS can support Alzheimer disease diagnosis in memory and visuo-spatial tests. Entropy.

[30] Keshmiri S., Sumioka H., Yamazaki R., Ishiguro H. (2019). Multiscale entropy quantifies the differential effect of the medium embodiment on older adults prefrontal cortex during the story comprehension: A comparative analysis. Entropy.

[31] Xu C., Xu C., Tian W., Hu A., Jiang R. (2019). Multiscale entropy analysis of page views: A case study of Wikipedia. Entropy.

[32] Shang H., Li F., Wu Y. (2019). Partial discharge fault diagnosis based on multi-scale dispersion entropy and a hypersphere multiclass support vector machine. Entropy.

[33] Siegenfeld A.F., Bar-Yam Y. (2020). An introduction to complex systems science and its applications. Complexity.

[34] Bar-Yam Y. (2003). Complexity of Military Conflict: Multiscale Complex Systems Analysis of Littoral Warfare; Report to Chief of Naval Operations Strategic Studies Group. https://citeseerx.ist.psu.edu/document?repid=rep1&type=pdf&doi=5df6f5cf23134345fc98008a6f933459b32f5021.

[35] Yin J., Su C., Zhang Y., Fan X. (2018). Complexity analysis of carbon market using the modified multi-scale entropy. Entropy.

[36] Nelson E. (1966). Derivation of the Schrödinger equation from Newtonian mechanics. Phys. Rev..

[37] Nelson E. (1979). Connection between Brownian motion and quantum mechanics. Proceedings of the Einstein Symposion Berlin: Aus Anlaß der 100. Wiederkehr Seines Geburtstages 25. bis 30. März 1979.

[38] Allen B., Stacey B.C., Bar-Yam Y. (2017). Multiscale information theory and the marginal utility of information. Entropy.

[39] Landau L.D. (1933). Electron Motion in Crystal Lattices. Phys. Z. Sowjetunion.

[40] Zabusky N.J., Kruskal M.D. (1965). Interaction of "solitons" in a collisionless plasma and the recurrence of initial states. Phys. Rev. Lett..

[41] Fleischer J.W., Segev M., Efremidis N.K., Christodoulides D.N. (2003). Observation of two-dimensional discrete solitons in optically induced nonlinear photonic lattices. Nature.

[42] Zaera R., Vila J., Fernandez-Saez J., Ruzzene M. (2018). Propagation of solitons in a two-dimensional nonlinear square lattice. Int. J. Non-Linear Mech..

[43] Liu M., Wei Z.W., Luo A.P., Xu W.C., Luo Z.C. (2020). Recent progress on applications of 2D material-decorated microfiber photonic devices in pulse shaping and all-optical signal processing. Nanophotonics.

[44] Feng T., Li X., Guo P., Zhang Y., Liu J., Zhang H. (2020). MXene: Two dimensional inorganic compounds, for generation of bound state soliton pulses in nonlinear optical system. Nanophotonics.

[45] Landau L.D. (1937). On the Theory of Phase Transitions. Zhurnal Eksperimental Teor. Fiz..

[46] Ginzburg V.L., Ginzburg V.L., Landau L. (2009). On the Theory of Superconductivity.

[47] Pekar S. (1946). Autolocalization of the electron in an inertially polarizable dielectric medium. Zhurnal Eksperimental Teor. Fiz..

[48] Landau L.D., Pekar S. (1948). Effective mass of a polaron. Zhurnal Eksperimental Teor. Fiz..

[49] Sio W.H., Giustino F. (2023). Polarons in two-dimensional atomic crystals. Nat. Phys..

[50] Kadanoff L.P. (1986). Fractals: Where’s the Physics?. Phys. Today.

[51] Mandelbrot B.B. (1983). The Fractal Geometry of Nature.

[52] Soljacic M., Segev M., Menyuk C.R. (2000). Self-similarity and fractals in soliton-supporting systems. Phys. Rev. E.

[53] Caticha A. (2001). Change, time and information geometry. AIP Conf. Proc..

[54] Caticha A. (2011). Entropic time. AIP Conf. Proc..

[55] Caticha A. (2021). Entropic Physics: Probability, Entropy, and the Foundations of Physics. https://www.albany.edu/physics/faculty/ariel-caticha.

[56] Gulevich D., Kusmartsev F. (2006). New phenomena in long Josephson junctions. Supercond. Sci. Technol..

[57] Gulevich D., Kusmartsev F. (2006). Perturbation theory for localized solutions of the sine-Gordon equation: Decay of a breather and pinning by a microresistor. Phys. Rev. B.

[58] Gulevich D.R., Kusmartsev F., Savel’ev S., Yampol’skii V., Nori F. (2008). Shape waves in 2D Josephson junctions: Exact solutions and time dilation. Phys. Rev. Lett..

[59] Kusmartsev F. (1984). On classification of solitons. Phys. Scr..

[60] Zakharov V.E. (1968). Stability of periodic waves of finite amplitude on the surface of a deep fluid. J. Appl. Mech. Tech. Phys..

[61] Zakharov V., Kuznetsov E. (1974). On three dimensional solitons. Zhurnal Eksperimental Teor. Fiz..

[62] Kusmartsev F. (1991). Multiphonon absorption of light in nonpolar crystals. Phys. Rev. B.

[63] Zakharov V.E. (1972). Collapse of Langmuir waves. Sov. Phys. JETP.

[64] Kusmartsev F., Rashba E. (1984). Symmetry Breaking in the Theory of Self-Trapping Barrier and in Allied Problems. Phys. Status Solidi (b).

[65] Kusmartsev F.V. (1989). Application of catastrophe theory to molecules and solitons. Phys. Rep..

[66] Novikov S., Manakov S.V., Pitaevskii L.P., Zakharov V.E. (1984). Theory of Solitons: The Inverse Scattering Method.

[67] Kusmartsev F., Rashba E. (1984). Self trapping from degenerate bands (spin S = 1) and related phenomena. Sov. Phys. JETP.

[68] Zakharov V., Musher S., Rubenchik A. (1985). Hamiltonian approach to the description of non-linear plasma phenomena. Phys. Rep..

[69] Kusmartsev F., Rashba E. (1983). Zh. eksper. teor. Fiz., Pisma 37, 106 (1983); CAS Soviet Phys. J. Exper. Theor. Phys. Lett. JETP Lett..

[70] Scott A. (1992). Davydov’s soliton. Phys. Rep..

[71] Damgaard Hansen S., Nygaard N., Mølmer K. (2021). Scattering of matter wave solitons on localized potentials. Appl. Sci..

[72] Lee M.H. (2017). Chemical potential, Gibbs–Duhem equation and quantum gases. Int. J. Mod. Phys. B.

[73] Coifman R.R., Wickerhauser M.V. (1992). Entropy-based algorithms for best basis selection. IEEE Trans. Inf. Theory.

[74] Santoro G.E., Martonák R., Tosatti E., Car R. (2002). Theory of quantum annealing of an Ising spin glass. Science.

[75] O’Hare A., Kusmartsev F.V., Kugel K.I. (2012). Stable forms of two-dimensional crystals and graphene. Phys. B Condens. Matter.

[76] O’Hare A., Kusmartsev F., Kugel K. (2012). A stable “flat” form of two-dimensional crystals: Could graphene, silicene, germanene be minigap semiconductors?. Nano Lett..

[77] O’Hare A., Kusmartsev F., Kugel K. (2009). Two-dimensional Ising model with competing interactions: Phase diagram and low-temperature remanent disorder. Phys. Rev. B.

[78] Kroese D.P., Brereton T., Taimre T., Botev Z.I. (2014). Why the Monte Carlo method is so important today. Wiley Interdiscip. Rev. Comput. Stat..

[79] Contreras-Reyes J.E., Quintero F.O.L., Wiff R. (2018). Bayesian modeling of individual growth variability using back-calculation: Application to pink cusk-eel (Genypterus blacodes) off Chile. Ecol. Model..

[80] Stauffer D., Aharony A. (2018). Introduction to Percolation Theory.

[81] Yakhot V., Orszag S.A. (1986). Renormalization group analysis of turbulence. I. Basic theory. J. Sci. Comput..

[82] Koch-Janusz M., Ringel Z. (2018). Mutual information, neural networks and the renormalization group. Nat. Phys..

[83] Schuld M., Sinayskiy I., Petruccione F. (2015). An introduction to quantum machine learning. Contemp. Phys..

[84] Carbone A., Jensen M., Sato A.H. (2016). Challenges in data science: A complex systems perspective. Chaos Solitons Fractals.

[85] Balanov A., Janson N., Postnov D., Sosnovtseva O. (2009). From Simple to Complex.

[86] Andreev A., Balanov A., Fromhold T., Greenaway M., Hramov A., Li W., Makarov V., Zagoskin A. (2021). Emergence and control of complex behaviors in driven systems of interacting qubits with dissipation. npj Quantum Inf..

[87] Seung H.S., Sompolinsky H., Tishby N. (1992). Statistical mechanics of learning from examples. Phys. Rev. A.

[88] Wang D., Tan D., Liu L. (2018). Particle swarm optimization algorithm: An overview. Soft Comput..

[89] Myint P.C., Gersten B.T., McClelland M.A., Nichols A.L., Springer H.K. (2018). Entropy maximization and free energy minimization of multiphase mixtures using particle swarm optimization. AIP Conf. Proc..

[90] Tanaka S. (2016). Diffusion Monte Carlo study on temporal evolution of entropy and free energy in nonequilibrium processes. J. Chem. Phys..

[91] Wissner-Gross A.D., Freer C.E. (2013). Causal entropic forces. Phys. Rev. Lett..

[92] Sánchez-Cañizares J. (2021). The free energy principle: Good science and questionable philosophy in a grand unifying theory. Entropy.

[93] Ahn K., Cong L., Jang H., Kim D.S. (2024). Business cycle and herding behavior in stock returns: Theory and evidence. Financ. Innov..

[94] Kusmartsev V., Drożdż M., Schuster-Böckler B., Warnecke T. (2020). Cytosine methylation affects the mutability of neighboring nucleotides in germline and soma. Genetics.

